# Supplementary material for: Molecular Detection of blaTEM and blaSHV Genes in ESBL-Producing Acinetobacter baumannii Isolated from Antarctic Soil
Source: Microorganisms. 2025 Feb 21;13(3):482. doi: 10.3390/microorganisms13030482 (PMC11945639; doi:10.3390/microorganisms13030482)
Supplement: Supplementary file 1 [file microorganisms-13-00482-s001.zip › Supplementary information_Table_S1_Figure_S1.pdf]

Supplementary Table S1 List of the primers used for detection of Antibiotic Resistance Genes (ARG)

Gene	Primer's name	Sequence (5'-3')	PCR product (bp)	Annealing (°C)	Reference
***bla*$_{TEM}$**	410F	GGTCGCCGCATACACTATTCTC	372	60	[1]
	781R	TTTATCCGCCTCCATCCAGTC			
***bla*$_{SHV}$**	287F	CCAGCAGGATCTGGTGGACTA	231		
	517R	CCGGGAAGCGCCTCAT			
***bla*$_{CTXM-1}$**	115F	GAATTAGAGCGGCAGTCGGG	588		
	702R	CACAACCCAGGAAGCAGGC			
***bla*$_{CTXM-2}$**	39F	GATGGCGACGCTACCCC	107		
	145R	CAAGCCGACCTCCCGAAC			
***bla*$_{CTXM-9}$**	16F	GTGCAACGGATGATGTTCGC	475		
	490R	GAAACGTCTCATCGCCGATC			
***bla*$_{CTXM-8/25}$**	533F	GCGACCCGCGCGATAC	186		
	718R	TGCCGGTTTTATCCCCG			
***bla*$_{KPC}$**	KPCfw	CGTCTAGTTCTGCTGTCTTG	798	55	[2]
	KPCrv	CTTGTCATCCTTGTTAGGCG			
***bla*$_{VIM}$**	VIMfw	GATGGTGTTTGGTCGCATA	390		
	VIMrv	CGAATGCGCAGCACCAG			
***bla*$_{NDM}$**	NDMfw	GGTTTGGCGATCTGGTTTTC	621		
	NDMrv	CGGAATGGCTCATCACGATC			
***bla*$_{OXA-48}$**	OXA48fw	GCGTGGTTAAGGATGAACAC	438		
	OXA48rv	CATCAAGTTCAACCCAACCG			

Reference:

1. Hoa Ly, T.; Tuyet Hoa, T.T.; Mong Huyen, H. Antibiotic Resistance and Molecular Characteristics of Extended-Spectrum Beta-Lactamase-Producing Escherichia Coli Isolated from Fish Pond. *Can Tho University Journal of Science* 2018, *54(8)*, 114, doi:10.22144/ctu.jen.2018.045.
2. Poirel, L.; Walsh, T.R.; Cuvillier, V.; Nordmann, P. Multiplex PCR for Detection of Acquired Carbapenemase Genes. *Diagn Microbiol Infect Dis* 2011, *70*, 119–123, doi:https://doi.org/10.1016/j.diagmicrobio.2010.12.002.

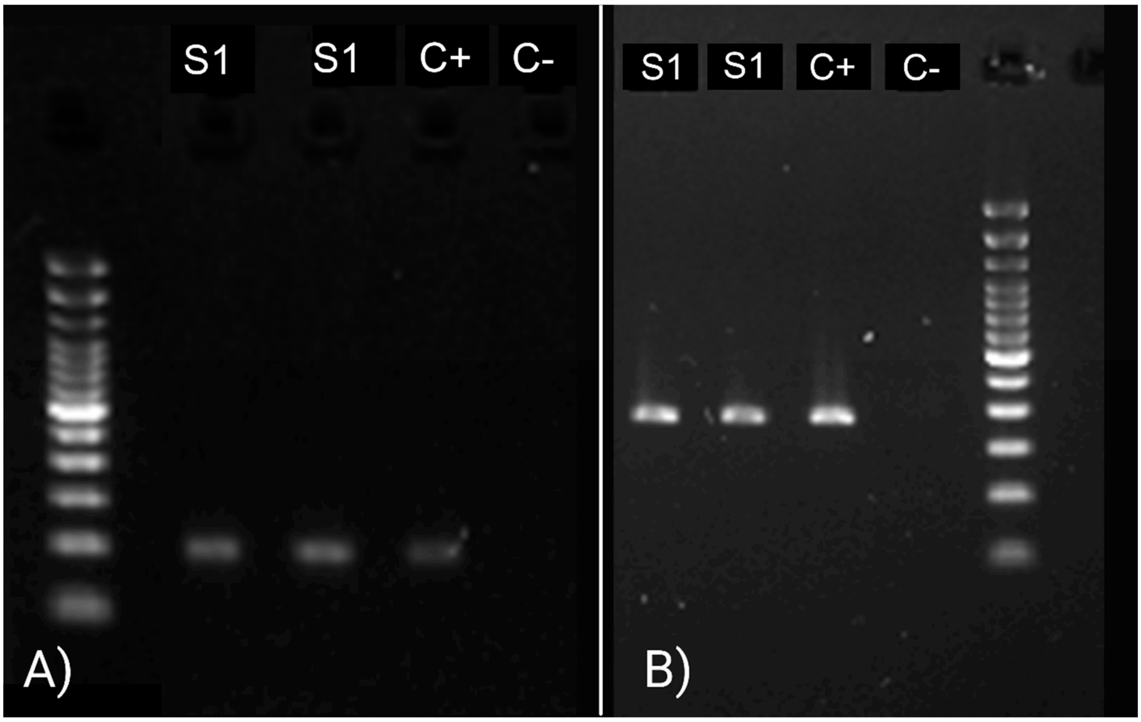

Supplementary Figure S1. Electrophoresis gels of PCR for *bla*$_{TEM}$ and *bla*$_{SHV}$ where in A) is presented a 231bp amplicon of *bla*$_{SHV}$ and in B) a 372bp amplicon of *bla*$_{TEM}$.
