# Supplementary figures and images for: Molecular Detection of blaTEM and blaSHV Genes in ESBL-Producing Acinetobacter baumannii Isolated from Antarctic Soil
Source: Microorganisms. 2025 Feb 21;13(3):482. doi: 10.3390/microorganisms13030482 (PMC11945639; doi:10.3390/microorganisms13030482)

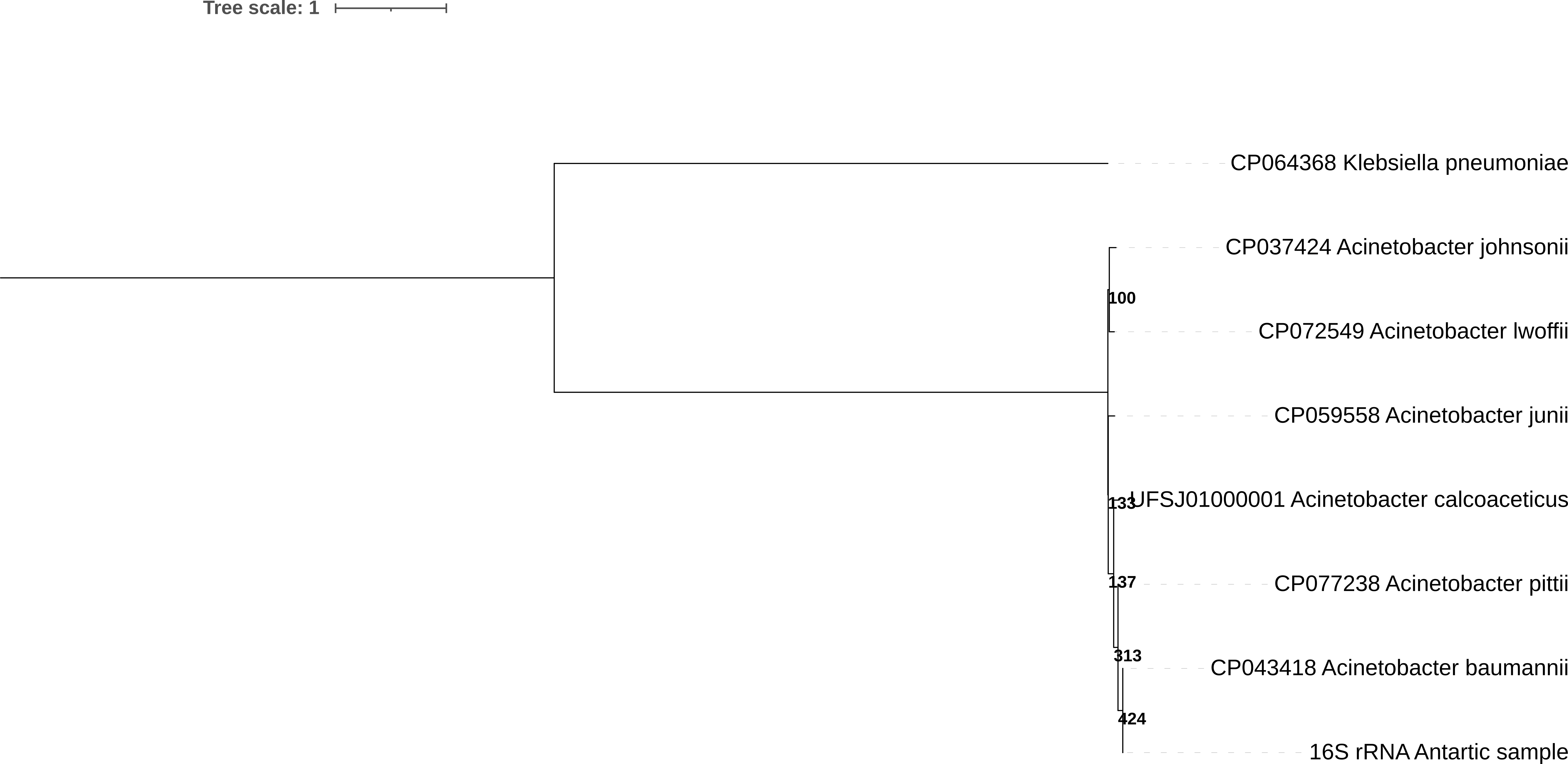

Supplement: Supplementary file 1 [file microorganisms-13-00482-s001.zip › Supplementary_Figure_2.png]

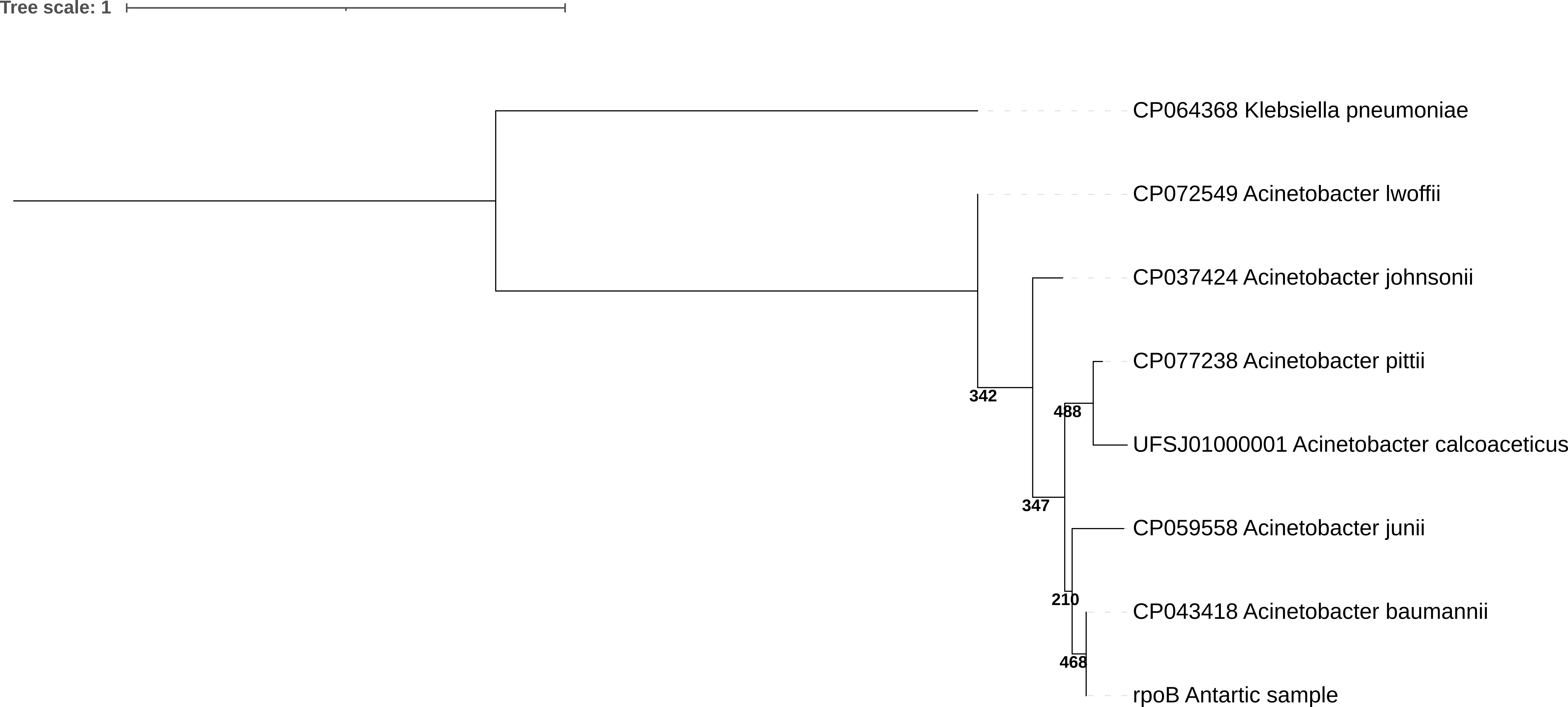

Supplement: Supplementary file 1 [file microorganisms-13-00482-s001.zip › Supplementary_Figure_3.png]
